# Cytokine Dynamics During Ustekinumab Induction as Predictors of Treatment Response in Crohn’s Disease: An Observational Study

**DOI:** 10.3390/biomedicines13112608

**Published:** 2025-10-24

**Authors:** Alejandro Mínguez, Beatriz Mateos, Marisa Iborra, Mariam Aguas, Guillermo Bastida, Alejandro Garrido, Elena Cerrillo, Sonia García, Lluís Tortosa, Inés Moret, Pilar Nos

**Affiliations:** 1 IBD Unit, Gastroenterology Department, La Fe University and Polytechnic Hospital, 46026 Valencia, Spain; 2 Inflammatory Bowel Disease Research Group, Health Research Institute La Fe (IIS La Fe), 46026 Valencia, Spain; bmateosa@gmail.com (B.M.);

**Keywords:** Crohn’s disease, ustekinumab, cytokines, biomarkers, therapeutic drug monitoring, treatment response, personalized medicine

## Abstract

**Background/Objectives**: Crohn’s disease (CD) is a chronic immune-mediated disorder with heterogeneous response to biologic therapies. Ustekinumab (UST), an anti-IL-12/23 monoclonal antibody, is effective in CD, but predictive biomarkers of treatment response remain lacking. This study aimed to investigate cytokine dynamics during UST induction and to evaluate their association with clinical and biochemical outcomes in an observational cohort of CD patients. **Methods**: We prospectively recruited 31 adult patients with moderate-to-severe active CD initiating UST therapy at a tertiary referral center. Peripheral blood and stool samples were collected at baseline and weeks 4, 8, and 16. UST trough concentrations, C-reactive protein (CRP), fecal calprotectin (FC), hemoglobin, albumin, and 13 serum cytokines (including IL-1β, IL-6, IL-8, IL-10, IL-12p70, IL-13, IL-17, IL-23, TNF-α, and OSM) were analyzed. Response was defined as a ≥70% reduction in FC at week 16, or, alternatively, CRP < 5 mg/L or a Harvey–Bradshaw Index < 3. **Results**: Eighteen patients (58%) achieved response at week 16. Responders showed significant reductions in FC, CRP, and disease activity, while non-responders exhibited limited biochemical improvement. Overall, UST induction was associated with a global decrease in proinflammatory cytokines, particularly TNF-α and IL-1β. Responders displayed distinct cytokine patterns, with higher IL-13 levels at week 8 and lower IL-8 concentrations at week 16 compared with non-responders. UST trough levels tended to be higher in responders, and inverse correlations were observed between drug concentrations and several cytokines, including IL-6, IL-8, IL-13, and IL-23. **Conclusions**: UST induction leads to measurable immunological changes in CD, with differential cytokine dynamics distinguishing responders from non-responders. These findings support the potential of cytokine signatures, in combination with therapeutic drug monitoring, as pharmacodynamic biomarkers to optimize personalized treatment strategies in CD.

## 1. Introduction

Crohn’s disease (CD) is a chronic and immune-mediated illness that affects the intestinal tract, leading to transmural inflammation and complications such as fistulas or stenosis. It is characterized by unpredictable periods of flare-ups and remission. Its complex etiology involves an intricate interplay of genetic predisposition, dysregulated immune responses and environmental factors [1,2]. Over the years, advancements in the understanding of the disease have led to the development of targeted therapies. First-line drugs include 5-aminosalicylates, corticosteroids and immunosuppressants [3]; however, they often fail to provide sustained remission and can be accompanied by significant side effects. Therefore, patients with moderate to severe CD often require biologic agents in follow-up, in many cases early after diagnosis. Some examples of these treatments include infliximab, adalimumab, vedolizumab, or ustekinumab (UST) [4,5].

UST is a human monoclonal antibody targeting the p40 subunit of interleukin (IL)-12 and IL-23 cytokine pathways [6]. UST has shown remarkable efficacy in inducing and maintaining remission in CD patients [7,8], positioning itself as a valuable second-line therapeutic alternative. While UST’s efficacy is well established, there remains a significant gap of knowledge concerning the intricate clinical and biochemical changes that transpire within patients undergoing treatment. Moreover, the precise determinants that underline an individual’s favorable or refractory response to this therapeutic agent remain elusive. Approximately, 42% of patients do not achieve clinical remission at week 26 of treatment, and fecal calprotectin (FC) does not return to normal in 46% of cases at week 52 [9,10]. Variability in treatment response prompts the need to identify predictive biomarkers that can guide personalized therapeutic strategies, ultimately leading to the accurate selection of the appropriate medication. Distinguishing responders from non-responders early in the treatment course would optimize patient outcomes and minimize unnecessary exposure to potentially ineffective interventions.

Cytokines emerge as possible biomarkers of treatment response, as they play a pivotal role in CD pathogenesis [11,12,13]. The imbalance between proinflammatory pathways and regulatory response leads to the excessive production of cytokines, particularly IFN-γ, TNF-α and IL-12 (Th1 pathway); IL-17 and IL-23 (Th17 pathway); IL-4 and IL-13 (Th2 pathway); IL-1β, IL-6 (innate immunity). Other promising attempts include using drug levels or clinical characteristics as potential indicators of treatment efficacy [14,15]. However, a lack of truly validated markers persists in the field.

In this observational study, conducted under routine clinical practice conditions, we aim to explore the alterations in the cytokine profile that occur during the induction period of UST in CD patients, with the intention of identifying key distinctions between individuals who respond to the treatment and those who do not. This will be achieved through the assessment of a range of clinical and biochemical parameters during the induction period, including serum cytokine levels as well as concentrations of UST.

## 2. Materials and Methods

### 2.1. Study Design and Patients

Adult patients (>18 years) with active CD, defined as FC > 250 μg/g [16], were prospectively recruited between 2019 and 2021, consecutively, at La Fe University and Polytechnic Hospital, a tertiary academic referral center in Spain. All participants provided written informed consent. All patients were of Caucasian ethnicity and recruited from the Mediterranean region of Spain.

The diagnosis of CD was established according to standard criteria [3]. UST was administered as an initial intravenous infusion of 6 mg/kg at baseline, followed by subcutaneous injections of 90 mg every 8 weeks, in accordance with the recommended treatment regimen [7]. All patients were UST-naïve and were monitored during the first 16 weeks of therapy.

### 2.2. Study Variables

The demographic and clinical data included in the study were sex, height, body weight, Montreal Classification of Crohn’s Disease (age at diagnosis, location and behavior), nº of prior anti-TNF-α exposure, other prior biologic exposure, concomitant drugs, smoking, previous surgery, need for UST intensification or reinduction and the Harvey–Bradshaw Index (HBI).

The biochemical profile of the participants was assessed by measuring C-reactive protein (CRP) levels, fecal calprotectin (FC) levels, hemoglobin levels (Hg), albumin levels (Alb) and UST concentrations. Thirteen serum cytokines involved in CD pathogenesis [16,17] were measured: interleukin (IL)-1β, IL-2, IL-6, IL-7, IL-8, IL-10, IL-12p70, IL-13, IL-17, IL-23, interferon gamma (IFN-γ), tumoral necrosis factor alpha (TNF-α) and oncostatin M (OSM).

### 2.3. Sample Collection and Measurement

To assess biochemical parameters of inflammation, we collected peripheral blood and stool samples prior to every UST administration at week 0, week 8 and week 16. In addition, an extra blood sample at week 4 was incorporated for the determination of drug levels. 

Biochemical parameters were measured in blood samples at the routine laboratory of the hospital. Fecal samples were collected and stored at −20 °C until use. FC concentrations were determined by the Clinical Analysis Department, using a quantitative enzyme-linked immunoassay (Calprest^®^, Eurospital, Trieste, Italy) in a Best 2000 fully automated plate enzyme-linked immunosorbent assay (ELISA). For the analysis of UST levels, LISA-TRACKER Ustekinumab (Theradiag^®^, Croissy-Beaubourg, France) was used in Dynex Technologies—DS2 ELISA.

For cytokine measurement, peripheral blood samples were also collected at week 0, 8 and 16. The serum was separated by gravity centrifugation at 3000 rpm for 10 min. The upper phase, containing the serum, was separated from the gel and stored at −80 °C until use. Cytokines were measured by Luminex technology (MAGPIX^®^) at each time point following the manufacturer’s instructions. Data was collected using Luminex xPONENT^®^ 4.2 software.

### 2.4. Definition of Primary Response

Treatment response was primarily defined based on the relative reduction in FC from baseline (week 0) to week 16. Patients achieving a ≥70% reduction in FC were classified as responders, whereas those with <70% reduction were considered non-responders. When FC measurements were missing at any of the key time points, biochemical or clinical remission criteria were applied as surrogate endpoints: CRP < 5 mg/L at week 16 or HBI < 3. This hierarchical approach aimed to minimize data loss while maintaining the validity of response classification.

### 2.5. Statistical Analysis

Descriptive statistical analysis for biochemical characteristics was performed using standard methods (mean, median and interquartile range). To understand how peripheral blood cytokines change due to UST, we measured the effect size using Cohen’s d coefficient (mean week 0—mean week 16/grouped standard deviation). Polynomial regression models were applied to explore potential non-linear associations between UST through concentrations and biochemical markers. The regression curves were fitted using least-squares estimation and displayed together with individual data points to illustrate the overall trend. Spearman’s coefficient was used to address the correlation between different parameters. The Mann–Withney test was used for cytokine and biochemical comparisons between responders and non-responders at each time point. A *p*-value lower than 0.05 was considered significant. All figures and analysis were performed using R version 4.2.0. 

### 2.6. Ethics

This project was approved on 12 July 2019 by the Research Ethics Committee for Medicines at La Fe University and Polytechnic Hospital, according to the Declaration of Helsinki and was registered with the code BBN-INF-2019-01. Written informed consent was obtained from all participants.

## 3. Results

### 3.1. Baseline Characteristics

In this study, a total of 31 patients were recruited. All patients’ demographics and clinical characteristics at baseline are presented in Table 1.

From this cohort, eighteen patients (58%) achieved treatment response by week 16, while thirteen (42%) did not respond to UST treatment. Non-responders were mainly diagnosed at an age above 40 years old (53.8%) and had an ileocolonic disease (53.8%). In contrast, most responders were diagnosed between ages 17 and 40 (61.1%) and had either ileal (38.9%) or colonic (38.9%) behavior. One third of patients (38.7%) received concomitant drugs at the start of UST. Corticosteroids were used only in responders, whereas immunosuppressants were more common among non-responders.

### 3.2. Biochemical Characteristics

The biochemical profile showed significant changes over the course of the first 16 weeks in patients who responded to UST (Table 2). There was a significant decrease in the HBI (*p* = 0.002), CRP (*p* = 0.012) and FC (*p* < 0.001) among responders. Non-responders only showed a significant decrease in the HBI (*p* = 0.032). Neither Hg nor Alb levels were significantly different in any of the groups (Figure S1). 

### 3.3. Evolution of Ustekinumab Levels and Correlation with Biochemical Profiles

**Figure 1:**
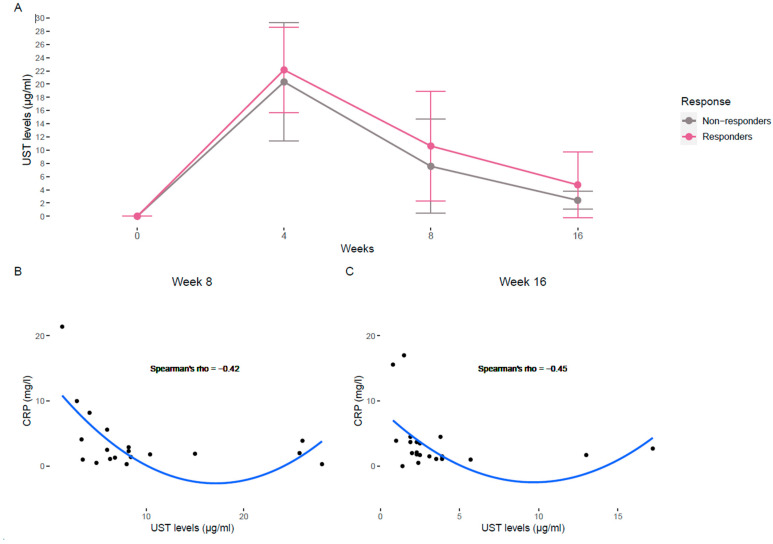
(**A**) Evolution of UST levels from baseline (week 0) to week 4, 8 and 16 of responders (pink) and non-responders (gray). Results are shown as the median concentration (points) and standard error (bars). (**B**) Negative Spearman correlation between CRP and UST levels at week 8 (all patients). (**C**) Negative Spearman correlation between CRP and UST levels at week 16 (all patients). A polynomial regression curve is represented in B and C plots. UST, Ustekinumab; CRP, C-reactive protein.

UST concentrations tend to be higher (non-significant) in responders throughout all the observed weeks (Figure 1A). UST intensification was performed in 13 patients (41.9%), consisting of either an additional intravenous reinduction dose or interval shortening to every 4 weeks. Among these, six patients achieved biochemical improvement, while the remainder did not respond despite increased drug exposure. UST trough levels tended to rise post-intensification, but this was not consistently associated with clinical benefit. Intensification was more frequent in non-responders, reflecting attempts to rescue treatment response. Indeed, when exploring the correlations between drug levels and biochemical variables, moderate negative spearman correlations of −0.42 and −0.45 were observed between UST levels and CRP at week 8 and week 16, respectively (Figure 1B,C), showing that CRP values decrease with increasing UST concentrations up to approximately 10 μg/mL, after which a plateau or slight upward trend is observed at higher concentrations. 

### 3.4. Serum Cytokines and Biochemical Profiles

#### 3.4.1. Overall Cytokine Behaviors

When analyzing the overall behavior of cytokines following UST induction, notable interactions with the drug were observed. Cohen’s *d* coefficient was used to calculate the effect size between week 0 and week 16. The general trend displayed a decrease in all cytokines except IL-7, which showed a marginal increase, while OSM remained unchanged during the weeks. The most substantial effect sizes were observed for TNF-α (Cohen’s *d* = −0.58 95% CI [−1.20, 0.04]) and IL-1β (Cohen’s *d* = −0.47 95% CI [−1.09, 0.15]), both showing a moderate decrease from week 0 to week 16 (Table 3). 

#### 3.4.2. Clinical, Biochemical and Cytokine Differences Among Responders and Non-Responders

**Figure 2:**
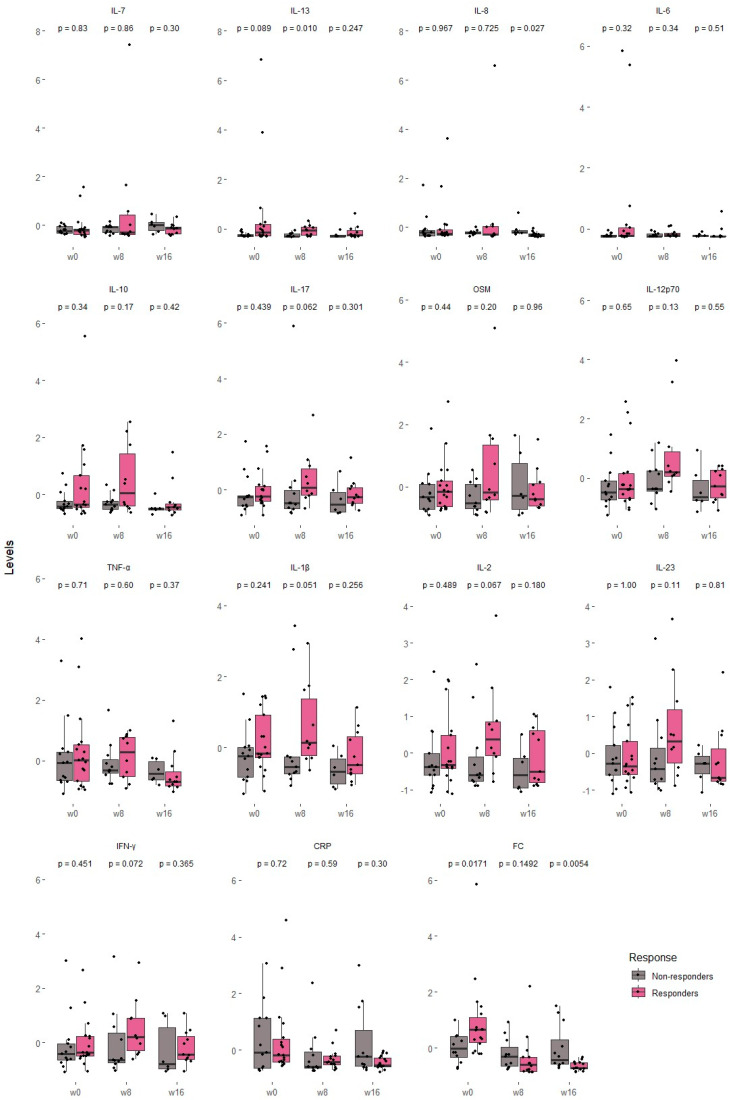
Comparison of cytokine serum levels between UST responders vs. UST non-responders before starting treatment (week 0) and during the induction period (week 8 and week 16). The depicted cytokine concentrations are scaled for visualization purposes. A Mann–Whitney test was used to determine statistical differences between responders and non-responders. CRP, C-reactive protein; OSM, Oncostatin M, FC, fecal calprotectin.

We compared cytokine concentrations, CRP and FC between responders and non-responders at specific time points (week 0, week 8 and week 16) (Figure 2). 

At baseline (week 0), responders showed higher FC concentrations than non-responders (*p* < 0.05), while no other variable reached statistical significance. By week 8, IL-13 levels were significantly higher in responders (*p* = 0.01), and IL-17, IL-1β, IL-2, and IFN-γ showed trends toward significance, with consistently higher levels in responders. At week 16, responders exhibited significantly lower concentrations of IL-8 (*p* < 0.05) and FC (*p* < 0.001). Notably, CRP, HBI, hemoglobin and albumin did not differ significantly between groups at any time point (Supplementary Figure S1).

### 3.5. Ustekinumab Levels and Cytokine Profiles 

We conducted a Spearman correlation analysis between cytokines and UST levels at both week 8 and week 16 (Table 4). At week 8, UST levels were inversely correlated with all cytokines except TNF-α, which showed a weak positive correlation (*ρ* = 0.19). The strongest inverse correlations at week 8 were observed for IL-6 (*ρ* = −0.45), IL-8 (*ρ* = −0.40), IL-13 (*ρ* = −0.39) and IL-23 (*ρ* = −0.38), indicating that higher UST levels were associated with lower cytokine levels. At week 16, IL-8 (*ρ* = −0.49) and IL-23 (*ρ* = −0.38) maintained a moderate inverse correlation, while OSM strengthened from a weak to a moderate inverse correlation (*ρ* = −0.40).

## 4. Discussion

In this real-world study in patients with moderate-severe CD, we have investigated the effect of UST induction on the profile of cytokines involved in the pathophysiology of the disease, also considering the influence of UST pharmacokinetics on the immune system. Our findings confirm the biochemical effectiveness of UST at week 16 with 58% biochemical remission and describe differential patterns of behavior from pro- and anti-inflammatory cytokines between responders and non-responders, with negative correlations between UST levels and the cytokines studied.

Both the effectiveness and safety of UST in patients with CD have already been extensively validated in pivotal studies [7,18] and in multicenter real-world studies [9,10,19]. Our results are in line with the outcomes of these studies, confirming UST effectiveness. However, as in previous series, there is interindividual variability in the response to treatment and we do not have validated biomarkers that allow us to predict treatment response [16,20]. In this context, the characterization of immunological profiles through the measurement of serum cytokines is a promising strategy. Multiple studies have already highlighted the central role of the IL-12/23-Th17 axis in the pathogenesis of CD, through cytokines such as IL-23, IL-17A, IL-22, TNF-α and OSM [6,17,21]. By inhibiting the IL-23 signaling pathway, UST reduces Th17 expansion and proinflammatory cytokine production, resulting in clinical and endoscopic improvement [7,11,18].

Analyzing serum cytokines, we observed an overall decrease in cytokine levels following UST induction—with TNF-α and IL-1β showing the most substantial reductions, except for IL-7, which increased slightly, and OSM, which was unchanged. The decrease in most ILs in our patient series is in line with other studies documenting decreases in the ILs studied, for example IL-22 [22], IL-23, INF-γ and TNF-α [6]. Previous studies analyzing the response to other biologic drugs found that in patients treated with IFX, higher titers of TNF-α, IL-7 and OSM were associated with a worse response to the drug [23]. Regarding OSM, from the IL-6 family, several studies postulate it as a cytokine that confers a suboptimal response to IFX and ADA [6,17], although there is still no robust evidence with UST [22,24].

Clinical predictors of non-response to UST, such as male sex, the presence of extraintestinal manifestations or the use of corticosteroids at the start of UST [25], as well as predictors of response, such as the uncomplicated phenotype or the absence of previous surgery, have been previously described [26]. Regarding the differences between patients with favorable and poor responses to treatment, we did not identify statistically significant baseline differences in the ILs studied. However, there was a trend toward higher levels of most of them before the start of UST in responders, findings in line with previous research [27]. Differences between responders and non-responders were apparent, with responders exhibiting higher levels of IL-13 at week 8, followed by a significant reduction in IL-8 at week 16. The identification of specific cytokine dynamics (e.g., IL-13 at week 8, IL-8 at week 16] under a robustly defined response framework may enhance the predictive value of these biomarkers for early treatment optimization with ustekinumab. It was reported that a higher level of IL-17A prior to UST treatment was associated with a good response [27]. We found a tendency to have higher levels of this IL at week 8 in the responder group. In contrast to our results, another study found that IL-6, INFγ and IL-22 decreased significantly in the responder group as potential predictors of response to UST, although in this case endoscopic remission was assessed and not biochemical remission as in our study [28]. However, lower IL-22 was not associated with clinical or biochemical remission in subsequent studies [22]. Finally, in the prospective study by Murate et al., a combination of low SES-CD and high TNF-α levels at the start of UST treatment in CD patients was associated with a better response to the drug [29].

In our series of patients, there was a tendency for higher drug levels in responders compared to non-responders throughout the induction of UST. The higher UST levels found in responders may be related to individual pharmacokinetic variability and greater drug exposure, which could have facilitated treatment response. Anti-ustekinumab antibodies are routinely tested in our center when UST levels are undetectable; however, this situation did not occur in any patient from our cohort. Even so, in both responders and non-responders, levels at weeks 4 and 8 were supratherapeutic for our laboratory (VN 1–4.5). Only at week 16 in the non-responder group did the levels go from supratherapeutic to in-range. These results support the previously established association between higher drug exposure in the induction period—higher blood levels—and clinical, biochemical and endoscopic improvement [8,14,22,30,31,32]. We identified a negative correlation of UST levels with CRP at weeks 8 and 16. Interestingly, the slight rise in CRP values observed at higher UST concentrations (around 20 μg/mL) may reflect a pharmacodynamic shift rather than a pharmacokinetic failure. Despite dose intensification and adequate or even supratherapeutic UST exposure, some patients may not achieve biochemical improvement, suggesting a lack of pharmacological efficacy at the target level. In contrast, at lower or intermediate UST concentrations (1–10 μg/mL), the inverse relationship between CRP and drug levels likely indicates a pharmacokinetic component, where suboptimal exposure results in insufficient inflammatory control. This finding suggests that higher systemic drug exposure is associated with greater biochemical improvement. However, given that UST concentrations were overall higher in responders compared with non-responders, it remains unclear to what extent the observed differences in treatment outcomes are driven by pharmacokinetic variability rather than true pharmacodynamic resistance. It would be advisable to perform therapeutic drug monitoring (TDM) in future biomarker studies [33]. Previous reports have demonstrated a positive correlation between serum and tissue UST concentrations [33], supporting the notion that systemic levels may, at least partially, reflect local drug exposure at the site of inflammation.

Moreover, correlation analyses between UST levels and cytokines at weeks 8 and 16 revealed an inverse relationship, particularly with IL-6, IL-8, IL-13, and IL-23, and OSM. IL-23, part of the Th17 inflammatory pathway, has been postulated as a key player in the pathophysiology of CD [11]. A previous study that looked at baseline ILs in patients treated with UST found that higher serum IL-23 prior to receiving the drug was associated with endoscopic healing at week 48 but did not look at the evolution of this after drug exposure [34]. Another study that also looked at IL-23 and UST levels, this time at week 16 of treatment, found no difference between responders and non-responders in both parameters. However, when studying the ratio of IL23/UST levels, it did discriminate between patients with and without biochemical response [35].

However, as our study has certain limitations, our results should be interpreted with caution. Given the limited sample size, our study should be interpreted as exploratory and hypothesis-generating. Larger, multicenter studies are warranted to validate these findings and better define cytokine-based biomarkers of response to UST. IL analysis was performed in peripheral blood (plasma) without a parallel study of inflamed tissue. However, Proietti et al. previously showed that tissue and serum levels of IL-23 in patients with CD treated with UST have similar behavior [35]. Our definition of response is based on the percentage reduction in FC, and in its absence, on CRP and/or HBI. The definition of treatment response was adapted from the STRIDE-II criteria [36]. Biochemical remission was defined as CRP levels within the normal range, while clinical remission was defined as HBI < 3. A ≥70% reduction in FC was selected as the threshold for biochemical response, representing a stricter criterion than the ≥50% reduction proposed in previous studies [37,38] and ensuring that patients with substantial improvement were adequately captured, even if FC normalization was not achieved. Since colonoscopy was not available in all patients in our study, we were unable to evaluate endoscopic healing. Finally, as this was an observational study, in some cases, the washout period may not have been sufficiently long to ensure complete clearance of prior biologic therapy, and some patients were receiving concomitant medications at the start of UST treatment. These factors could have influenced the baseline immune profile and cytokine expression.

In conclusion, this study provides novel evidence on the immunomodulatory effect of UST in patients with refractory CD in routine clinical practice, identifying differential IL patterns between UST responders and non-responders, thus suggesting its potential as a pharmacodynamic biomarker. Our findings suggest that serum biochemistry and cytokine profiles may help identify early, within the first 8–16 weeks of therapy, those patients at risk of non-response to ustekinumab. This approach could support timely treatment optimization and personalized management strategies in CD. These results also reinforce the value of the combination of therapeutic drug monitoring and immunological profiling as a strategy to optimize decisions in CD. However, given the small sample size and the exploratory nature of the study, the results should be interpreted with caution and warrant confirmation in larger, multicenter cohorts.

## Figures and Tables

**Table 1:** Baseline demographic and clinical characteristics of total (*n* = 31), responders (*n* = 18) and non-responders (*n* = 13) patients. Values of categorical variables are presented as numbers (percentage) and continuous variables are presented as medians (interquartile range, IQR).

Variable	Total (*n* = 31)	Responders (*n* = 18)	Non-Responders (*n* = 13)	*p*-Value
Sex, *n* (%)				1
Male	19 (61.3%)	11 (61.1%)	8 (61.5%)	
Female	12 (38.7%)	7 (38.7%)	5 (38.5%)	
Height (cm)	170 (165–177)	169 (165–179)	170 (165–175)	
Body weight (kg)	70 (62–82)	70 (62–81)	71 (66–80)	
Age at diagnosis, *n* (%)				0.213
A1	4 (12.9%)	3 (16.7%)	1 (7.7%)	
A2	16 (51.6%)	11 (61.1%)	5 (38.5%)	
A3	11 (35.5%)	4 (22.2%)	7 (53.8%)	
Location, *n* (%)				0.051
L1	10 (32.3%)	7 (38.9%)	3 (23.1%)	
L2	8 (25.8%)	7 (38.9%)	1 (7.7%)	
L3	10 (32.8%)	3 (16.7%)	7 (53.8%)	
L1 + L4	3 (9.7%)	1 (5.6%)	2 (15.4%)	
Behaviour, *n* (%)				0.753
B1	21 (67.7%)	12 (66.7%)	9 (69.2%)	
B2	4 (12.9%)	3 (16.7%)	1 (7.7%)	
B3	6 (19.4%)	3 (16.7%)	3 (23.1%)	
Perianal disease, *n* (%)	6 (19.4%)	3 (16.7%)	3 (23.1%)	
*n* of prior anti-TNF-α exposure, *n* (%)				0.393
0	4 (12.9%)	2 (11.1%)	2 (15.4%)	
1	18 (58.1%)	9 (50.0%)	9 (69.2%)	
2	9 (29.0%)	7 (38.9%)	2 (15.4%)	
Other prior biologic exposure, *n* (%)				0.245
0	28 (90.3%)	15 (83.3%)	13 (100%)	
1	3 (9.6%)	3 (16.7%)		
Concomitant drugs at the start of ust, *n* (%)				0.027
Corticosteroids	5 (16.1%)	5 (27.8%)		
Immunosuppressants	7 (22.6%)	2 (11.1%)	5 (38.5%)	
Smoking, *n* (%)				1
Yes	10 (32.3%)	6 (33.3%)	4 (30.8%)	
No	12 (38.7%)	7 (38.9%)	5 (38.5%)	
Ex	9 (29.0%)	5 (27.8%)	4 (30.8%)	
Surgery, *n* (%)	15 (48.4%)	8 (44.4%)	7 (53.8%)	
Need ust intensification/ reinduction, *n* (%)	13 (41.9%)	6 (33.3%)	7 (53.8%)	

**Table 2:** HBI and biochemical characteristics of responders and non-responders at weeks 0, 8 and 16. Data are shown as the median (IQR). *p*-values are given for differences between weeks within each group.

Variable	Responders				Non-Responders			
	Week 0	Week 8	Week 16	*p*-Value	Week 0	Week 8	Week 16	*p*-Value
HBI	5.12 5 (4–7)	2.44 2 (0.75–4)	1.75 1 (0–3.25)	0.002	4.15 5 (2–6)	2.27 2 (0–4)	1.31 1 (0–2)	0.032
CRP (mg/L)	7.83 4.15 (2.48–7.93)	3.21 2.5 (1.85–3.85)	2.36 1.7 (1.4–3.5)	0.012	7.7 4.8 (1–12.9)	4.28 1.4 (1–4.8)	6.95 3.7 (1.45–10.05)	0.55
FC (µg/g)	1906.4 1623 (1144–2098)	564.3 296.6 (53.3–586.5)	246 177.5 (142.5–369.7)	2.40 × 10^−6^	944.4 928.1 (575.2–1337.8)	709.9 620 (249.4–956.5)	911.6 493 (304.8–1240)	0.47
Hemoglobin (g/dL)	14.33 14.3 (12.9–15.8)	14.68 14.7 (13.5–16)	14.25 14.5 (13.5–15.6)	0.76	13.68 13.4 (13.1–14.2)	13.81 13.8 (13.4–14.8)	13.66 13.8 (13.4–14.7)	0.9
Albumin (g/dL)	4.29 4.5 (3.9–4.7)	4.407 4.45 (4.2–4.5)	4.45 4.5 (4.4–4.6)	0.52	4.24 4.3 (4.1–4.5)	4.31 4.3 (4.2–4.5)	4.45 4.5 (4.35–4.55)	0.32
UST levels (µg/mL)		10.627 8.2 (5.6–12.7)	4.75 2.8 (2.37–3.9)			7.55 6 (3.85–7.7)	2.41 2.1 (1.9–2.3)	

HBI, Harvey–Bradshaw Index; CRP, C-reactive protein; FC, fecal calprotectin; UST, ustekinumab.

**Table 3:** Effect size measured by Cohen’s d coefficient of the overall differences between week 0 and week 16 of each cytokine. A negative value means a decrease, while a value closer to 1 denotes larger differences.

Cytokine	Cohen’s d	Lower CI	Upper CI
TNF-α	−0.58	−1.20	0.04
IL-1β	−0.47	−1.09	0.15
IL-10	−0.38	−1.00	0.24
IL-8	−0.37	−0.99	0.24
IL-6	−0.33	−0.95	0.28
IL-13	−0.33	−0.94	0.29
IL-17	−0.24	−0.85	0.38
IL-2	−0.20	−0.82	0.41
IL-12p70	−0.20	−0.81	0.41
IL-23	−0.19	−0.80	0.43
INF-Γ	−0.18	−0.79	0.43
OSM	0	−0.61	0.61
IL-7	0.04	−0.57	0.65

CI, confidence interval; OSM, Oncostatin M.

**Table 4:** Spearman correlation between each cytokine and UST levels at week 8 and week 16. A negative value means an inverse correlation, while a value closer to 1 denotes larger correlation.

	Spearman Coefficient	
Cytokines	w8	w16
IL-6	−0.45	−0.22
IL-8	−0.40	−0.49
IL-13	−0.39	−0.08
IL-23	−0.38	−0.38
IL-2	−0.30	−0.26
IL-1β	−0.25	−0.34
IL-12p70	−0.23	−0.31
INF-γ	−0.23	−0.29
OSM	−0.18	−0.40
IL-17	−0.09	−0.12
IL-10	−0.08	−0.21
IL-7	−0.05	−0.23
TNF-α	0.19	−0.31

OSM, Oncostatin M.

## Data Availability

The original contributions presented in this study are included in the article/Supplementary Material. Further inquiries can be directed to the corresponding author.

## Supplementary Materials

The following supporting information can be downloaded at https://www.mdpi.com/article/10.3390/biomedicines13112608/s1.

## Abbreviations

The following abbreviations are used in this manuscript:

Alb	Albumin
CD	Crohn’s disease
CRP	C-reactive protein
FC	Fecal calprotectin
HBI	Harvey–Bradshaw Index
Hg	Hemoglobin
IBD	Inflammatory bowel disease
IL	Interleukin
INFγ	Interferon gamma
IQR	Interquartile range
OSM	Oncostatin M
TDM	Therapeutic drug monitoring
TNF-α	Tumor necrosis factor alpha
UST	Ustekinumab
