# Supplementary figures and images for: Cytokine Dynamics During Ustekinumab Induction as Predictors of Treatment Response in Crohn’s Disease: An Observational Study
Source: Biomedicines. 2025 Oct 24;13(11):2608. doi: 10.3390/biomedicines13112608 (PMC12650091; doi:10.3390/biomedicines13112608)

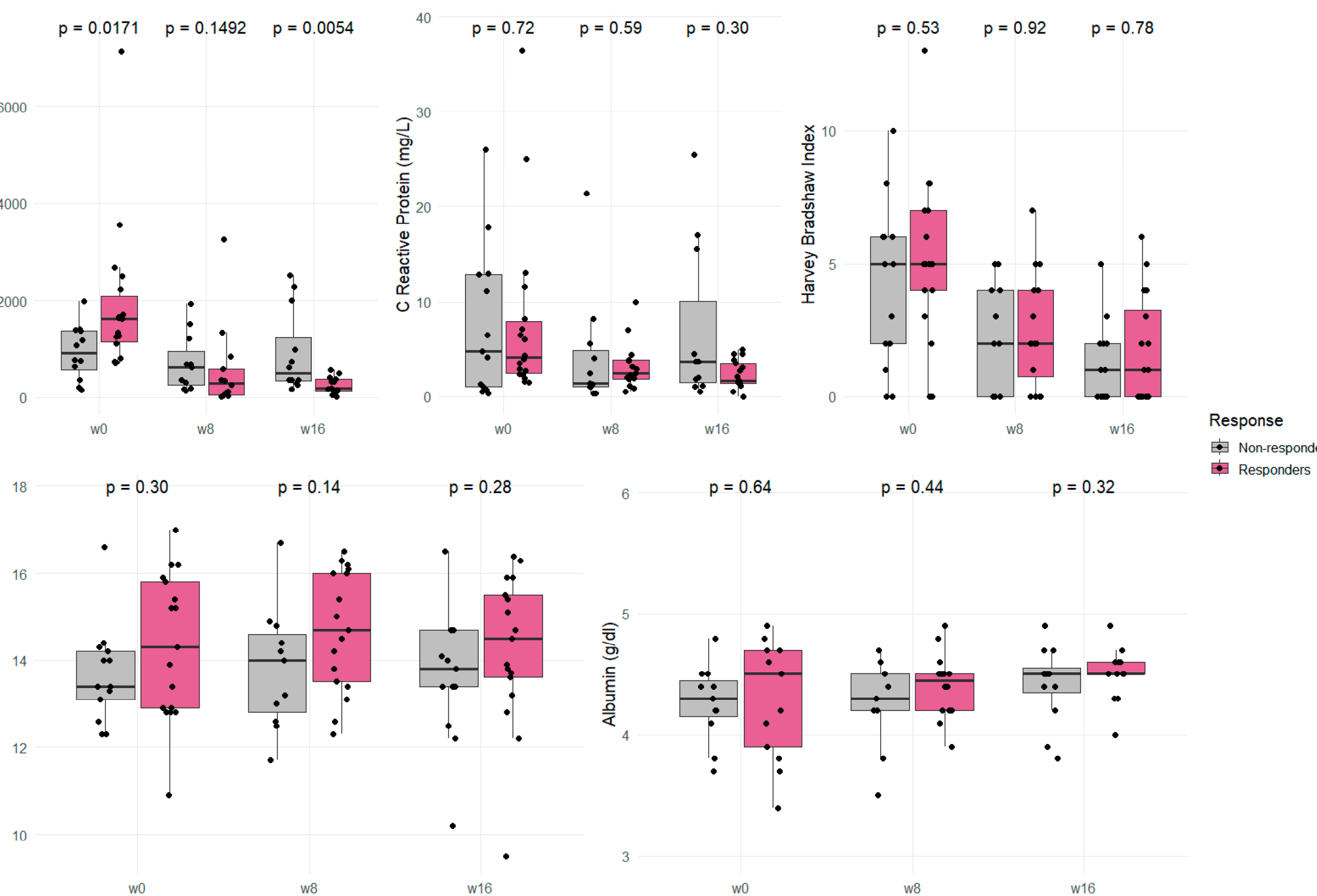

Supplement: Supplementary file 1 [file biomedicines-13-02608-s001.zip › biomedicines-3912379-supplementary.pdf]
